# Hispanic/Latinos and non-Hispanic whites’ childhood cancer survivors and parents: a dyadic analysis of coping resources and mental health

**DOI:** 10.1007/s11764-023-01339-8

**Published:** 2023-02-28

**Authors:** Carol Y. Ochoa, Junhan Cho, Kimberly A. Miller, Lourdes Baezconde-Garbanati, Randall Y. Chan, Albert J. Farias, Joel E. Milam

**Affiliations:** 1 Department of Population and Public Health Sciences, Keck School of Medicine, University of Southern California, N. Soto Street, 3rdFloor, Room 312-30, Los Angeles, CA 90032, USA; 2 Department of Radiation Medicine and Applied Sciences, University of California San Diego, San Diego, CA, USA; 3 Department of Pediatrics, Keck School of Medicine, University of Southern California, Los Angeles, CA, USA; 4 Department of Epidemiology and Biostatistics, Department of Medicine, University of California, Irvine, Irvine, CA, USA

**Keywords:** Childhood cancer survivors, Parents, Coping resources, Mental health

## Abstract

**Purpose:**

While limited, dyadic research demonstrates the interdependent relationship between the health and adjustment after treatment between cancer survivors and caregivers. We examined interrelationships between coping resources and mental health among childhood cancer survivors (CCS)–parent dyads.

**Methods:**

One hundred sixty CCS-parent dyads from the Project Forward pilot study completed validated questions assessing social support, religiosity, spirituality, depressive symptoms, and perceived stress. Bidirectional associations were identified with path analysis utilizing the actor-partner interdependence model (APIM). We used a multigroup approach to test for the moderating effects by Hispanic ethnicity on these relationships.

**Results:**

Mean age of CCS was 20 years old, 51% female, 30% diagnosed with leukemia, and mean of 7 years from diagnosis. The mean age of parents was 49 years old and 89% were mothers. For both CCS and parents, perceived social support was inversely associated with their depressive symptoms and perceived stress (e.g., actor effects). Parents’ social support was not significantly associated with CCS’s depressive symptoms and stress. However, higher perceived social support by the CCS was inversely associated with parents’ depressive symptoms (*β* = −0.202, *p* < 0.01) and perceived stress (*β* = −0.164, *p* < 0.05) (e.g., partner effects). Additional actor effects were observed between spirituality, religiosity, and depressive systems when we explored the moderating effects of Hispanic ethnicity.

**Conclusion:**

Partner effects of social support among CCS-parent dyads may influence psychological distress.

**Implication for Cancer Survivors:**

Our findings on parent–child associations between social support and psychosocial well-being imply that survivorship care can be enhanced when the social support needs of both survivors and their parents are addressed together.

## Background

More than 500,000 childhood cancer survivors (CCS) live in the USA, of whom their parents are the primary caregivers throughout their cancer journey [1]. CCS experience significant psychological distress years after post-treatment [2-5]. Brinkman et al. explored patterns of psychological distress in the longitudinal multi-site Childhood Cancer Survivor Study (CCSS) cohort [2]. They found subgroups of survivors with persistent symptoms during long-term survivorship and latent symptoms that emerged up to 13 years later. Similarly, CCS parents are also at risk for experiencing psychological effects such as posttraumatic stress symptomology (PTSS) and posttraumatic stress disorder (PTSD) [6-8], emotional distress [8, 9], depressive symptoms, and anxiety [7, 8, 10]. Furthermore, non-white minority CCS and parents experience greater psychological distress and impaired quality of life than non-Hispanic white populations [11-13].

A bidirectional relationship has been found, where a childhood illness such as a cancer diagnosis is influenced by characteristics associated with their illness and their family and social environment such as medical support they receive [14]. However, literature is sparse in assessing the bidirectional associations between young adult survivors of childhood cancer and parents. One study among CCS-parent dyads demonstrates that parents’ depressive symptoms negatively affect cancer survivors’ report of family cohesion [15], while greater cancer patients’ subjective illness severity is associated with greater caregivers’ PTSS [16]. The dyadic literature between adult survivors and spousal caregivers suggests positive reciprocal relationships for psychosocial adjustment exist beyond mental health “spillover effects” [17, 18]. For instance, higher levels of the spiritual well-being of adult survivors or caregivers are associated with better mental and physical health for both [18]. These findings suggest that CCS and parents’ well-being are interrelated and that dyadic effects within this population should be further explored.

These reciprocal relationships may be salient to explore among positive psychosocial adjustments, specifically between coping resources and mental health outcomes. Social support, spirituality, and religiosity are coping resources that can enhance mental well-being for both CCS and parents throughout the cancer trajectory [7, 19-23]. However, no research to date has examined if there are bidirectional relationships for how these coping resources are associated with psychological distress in CCS-parent dyads. Such relationships may provide insight into whether coping resources alleviate dyadic psychological distress. Furthermore, Hispanic/Latino CCS-parent dyads experience a greater degree of psychological distress [11, 24, 25]; yet are an understudied population with unique cultural values and beliefs that could contribute to their coping resources (e.g., perceived social support and their experiences with spirituality and religiosity) [26]. This study attempts to fill gaps in the dyadic context and expand the literature by exploring if Hispanic ethnicity plays a role in this relationship.

We presume that within a CCS-parent dyad, outcomes of interest are correlated with and affect each other. Utilizing the Actor-Partner Interdependence Model (APIM) framework where an individual (e.g., “actor effect”) and their dyadic partner can simultaneously affect (e.g., “partner effect”) the outcome of interest [27-29], we addressed three study aims. First, we examined the actor and partner effects of coping resources on depressive symptomology among parent–child dyads. Second, we examined if the coping resources of actor and partner effects would be reciprocally associated with their own and partner’s perceived stress. For both these aims, we hypothesized that there would be significant actor and partner associations of greater coping resources of parents and CCS being related to lower levels of depressive symptoms and perceived stress. Finally, we examine a nondirectional exploratory aim of whether Hispanic ethnicity has moderating effects on the association between coping resources on depressive symptoms and perceived stress on dyads.

## Methods

### Participant

Participants were from the Project Forward pilot study, a cross-sectional study that used population-based survey methods to recruit 160 parent–child dyad respondents [30]. CCS diagnosed at age < 18 years and were treated at either of two children hospitals: Children’s Hospital Los Angeles or Miller Children’s Hospital in Long Beach, were selected from the SEER cancer registry for Los Angeles County. Survivors met the inclusion criteria if they had (1) a diagnosis of any cancer except for Hodgkin’s lymphoma (these survivors were participating in a different study); (2) were diagnosed between 2000 and 2007; and (3) were able to read and write in English and/or Spanish to complete the survey.

### Procedures

The standard recruitment procedure for CCS and parents included two methods (detailed previously) [31]. If CCS were < 18 years at the time of the survey, both the parent and survivor were invited to participate; otherwise, the CCS was directly invited and subsequently asked for parental contact information and permission. Informed consent was obtained from both CCS and parents. Upon completing the survey, both received a $20 gift card for participating. All study procedures were approved by the California Committee for the Protection of Human Subjects, California Cancer Registry, and by human subject research committees at the University of Southern California, Children’s Hospital of Los Angeles, and Miller Children’s Hospital.

#### Measures

A standard dyadic design was used for this study at the time of data collection in 2009. Only one parent and CCS participated in the study, and both members were measured on the same independent and dependent variables.

#### Independent variables (coping resources selected based on theoretical and existing literature)

Social support — single-item measure of social support (SIMSS), a validated predictor of morbidity strongly associated with the composite social support index, was used [32]. It asked participants, “how many people do you have that you can count on for help when you need them, such as, to give rides to the hospital or store, or to help if you are sick?” Response options range from 0 = none, 1 = 1–2, to 2 = 3 or more, with higher scores indicating greater social support.

Religiosity/spirituality — two questions were asked about aspects of religiosity and spirituality. The first question assessed religiosity by asking the “frequency of religious service attendance,” and response options range from 0 = never, 1 = every few years, 2 = several times a year, 3 = 2–3 times per month, to 4 = at least once per week, with higher scores indicating a greater frequency of religious service attendance. The second question assessed spirituality by asking participants about the “importance of religion or spirituality,” and response options ranged from 0 = not important, 1 = somewhat important, to 2 = very important, with higher scores indicating greater importance of religion or spirituality.

#### Dependent variables

Depressive symptoms — with the validated 20-item Center for Epidemiological Studies Depression Scale (CES-D) [33], participants are asked to report the frequency of depressive symptoms that occurred during the past week using a 4-point Likert scale ranging from (0) none of the time to (3) all of the time. The present analysis used the total sum score, with higher scores indicating greater depressive symptoms. Cronbach’s alpha in this dyad sample was 0.84 for parents and 0.92 for CCS.

Perceived Stress Scale — stress was assessed using the validated 4-item version of the Perceived Stress Scale (PSS) [34]. Participants were asked the extent to which they felt or thought about each statement in the past month, with responses ranging from 0 = never to 4 = very often. Items were summed to create a total perceived stress score, with higher scores indicating more stress. Cronbach’s alpha in this dyad sample was 0.71 for parents and 0.54 for CCS.

#### Covariates

Demographic and clinical information was obtained from self-report and cancer registry data. CCS’s current age at the survey was used as a continuous variable. Treatment intensity was calculated using the Intensity of Treatment Rating Scale 2.0 (ITR-2) [35], a 4-level validated scale ranging from 1 = least intensive treatment (surgery only) to 4 = most intensive, from cancer registry data and medical chart review. Parent Hispanic ethnicity was self-reported in the survey. Additional potential covariates were also assessed, including parents’ primary language, parental education, socioeconomic status, CCS time since diagnosis, and whether CCS lived with parents. Based on the model fit indices and the literature that previously found an association between these covariates and quality of life outcomes [11, 25], the primary analysis models included three selected covariates: age at survey, treatment intensity, and parent Hispanic ethnicity.

#### Statistical analysis

SAS version 9.4 was used for data cleaning and to conduct descriptive analysis [36]. Descriptive statistics were conducted to compare demographic characteristics between CCS and parents using *t*-tests for continuous variables and chi-square for categorical variables. Pearson’s correlation coefficient was used to evaluate non-independence between the dyadic member’s scores on the coping resources predictor (social support, religiosity, and spirituality), covariates (age of CCS, treatment intensity, and parent Hispanic ethnicity), and dependent outcomes (depressive symptoms and perceived stress). We used Cohen’s criterion, where 0.5 is a large correlation, 0.3 is a medium correlation, and 0.1 is a small correlation [37].

Path analyses of APIM in Mplus version 8 were used to model the reciprocity of parent-CCS dyadic relationships and examine our hypotheses [38]. The full information maximum likelihood estimation method was used, which includes all dyad data in the analyses as long as one individual responds to the respective outcome. Due to theoretical consideration, APIM for distinguishable dyads was used to implement all models, which means path analysis results would give us two equations—one for each person within the dyads (e.g., parents and CCS) [39]. Two separate actor-partner models were conducted based on our two dependent variables (depressive symptoms and perceived stress), and all three independent variables (social support, religiosity, and spirituality) were entered into each model. Supplemental Figs. 1 and 2 illustrate the specification of our two models.

Several models were estimated, and model fit was statistically evaluated using the criterion described below. First, we examined our two models without controlling for covariates and found that our model fit was poor. In these APIM models, both actor and partner effects are examined simultaneously while controlling for variance explained by the partner. Second, we tested whether adding all potential covariates (see “Measures”) to these two models demonstrated a better model fit. Next, we used the model modification indices results from these models to determine which additional covariate pathways were significantly associated with the key study variables and improved our model fit. Based on the procedure, covariates in the final model were selected.

In our final models, both actor and partner effects were examined simultaneously while controlling for variance explained by the partner. Additionally, we included our covariates and four correlational paths across covariates; these were added to improve the model fit indices. Finally, the moderating effects of Hispanic ethnicity were tested using the multigroup approach, allowing parameter estimates to vary among Hispanics or non-Hispanic dyads. We calculated standardized coefficients (*β*) and used a *p*-value < 0.05 (two-tailed) to determine statistical significance.

The overall model fit was assessed using chi-square statistic (*χ*^2^), degree of freedom (df), a root mean square error of approximation (RMSEA), the standardized root mean residual (SRMR), Tucker-Lewis index (TLI), and comparative fit index (CFI) [40]. Adequate fit for a specified model to the data requires a non-significant chi-square statistic, RMSEA of less than or equal to 0.06, SRMR value of less than 0.08, TLI greater than 0.90, and CFI greater than 0.95.

## Results

### Sample characteristics

Selected demographic characteristics of dyads by Hispanic ethnicity are shown in Supplemental Table 1. Hispanic and non-Hispanic CCS were on average 20 years old (standard deviation = 2.85), diagnosed 7 years ago, and about one-third were diagnosed with leukemia. Non-Hispanic CCS had higher levels of education, with 55% reporting greater than high school education, compared to 34% of Hispanic CCS. On average, Hispanic parents were younger than non- Hispanic parents (47 vs. 52 years old). Most Hispanic parents had less than a high school education (57% vs. 6%) and the majority were in the low socioeconomic status category (74% vs. 12%) compared to non-Hispanic parents.

Summary statistics of the study variables, including means, standard deviations, and Pearson’s correlation coefficients, are provided in Supplemental Table 2. Parents reported higher levels of spirituality and religiosity than CCS (*p*’s < 0.001). CCS reported having more social support than parents (*p* = 0.0006). There were no differences in scores of depressive symptoms and perceived stress between parents and CCS. Bivariate analyses demonstrated significant medium to large correlations between CCS and parent reported study variables, both positive (*r* =0.18 to 0.78) and negative (*r* = −0.16 to −0.33). Additionally, we found significant differences based on Hispanic ethnicity (see Supplemental Table 3). Hispanic parents reported lower levels of social support, higher levels of religiosity and spirituality, and greater scores of depressive symptoms than non-Hispanic parents (all *p* < 0.05), while Hispanic CCS reported higher spirituality levels than non-Hispanic CCS (*p* < 0.05).

### Actor and partner effect models

**Fig. 1:**
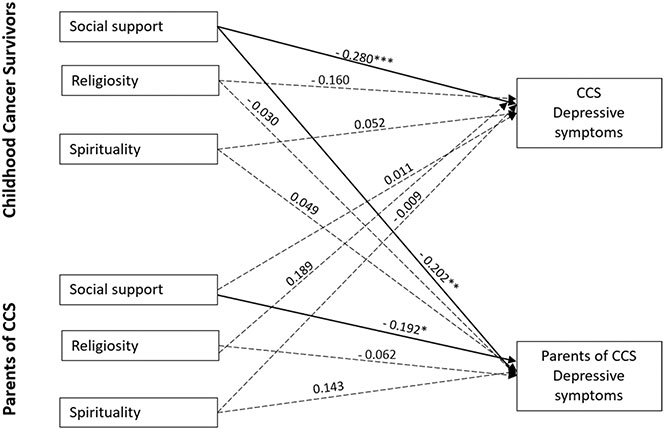
APIM model of social support, religiosity, and spirituality on CCS and parent depressive symptoms Note. CCS: childhood cancer survivors Figure values are standardized regression coefficients while controlling for CCS age, treatment intensity, and parent Hispanic ethnicity; Solid lines represent significant estimates and dashed lines represent statistical non-significant estimates. ***P < .001. **P < .01. *P < .05. ^†^P <.10 Error covariance estimates have been omitted for ease of presentation.

We first modeled the effect of social support, spirituality, and religiosity on self-reported depressive systems in the parent-CCS dyad using APIM (see Fig. 1). This model provided an excellent fit to the sample data, *χ*^2^ = 6.011, df = 8, CFI = 1.000, TLI = 1.109, RMSEA = 0.000 (90% CI = 0.000, 0.076), and SRMR = 0.023. There was a significant actor effect observed such that CCS perceived social support from others was negatively associated with their depressive symptoms (*β* = −0.280, *p* < 0.001) after controlling for confounding effects of all other variables (especially their partner’s effects: parents’ perceived social support). Additionally, parents’ perceived social support from others was negatively associated with their depressive symptoms (*β* = −0.192, *p* < 0.01). CCS perceived social support from others was negatively associated with their parents’ depressive symptoms (*β* = −0.202, *p* < 0.01). The corresponding partner effect from parent to CCS was not statistically significant when we simultaneously adjusted for actor effects. Furthermore, we found no significant actor or partner effect from spirituality and religiosity with depressive symptoms for both CCS and parents.

**Fig. 2:**
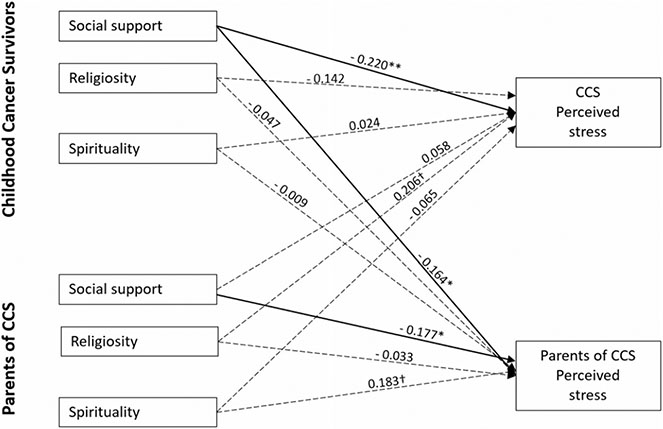
APIM model of social support, religiosity, and spirituality on CCS and parent perceived stress Note. CCS: childhood cancer survivors Figure values are standardized regression coefficients while controlling for CCS age, treatment intensity, and parent Hispanic ethnicity; Solid lines represent significant estimates and dashed lines represent statistical non-significant estimates. ***P < .001. **P < .01. *P < .05. ^†^P <.10. Error covariance estimates have been omitted for ease of presentation.

The second model was identical to the first, except that perceived stress was the outcome of interest (see Fig. 2). This model also provided an excellent fit to the sample data, *χ*^2^ = 6.044, df = 8, CFI = 1.000, TLI = 1.279, RMSEA = 0.000 (90% CI = 0.000, 0.076), and SRMR = 0.024. Similarly, we found two actor effects such that CCS perceived social support from others was negatively associated with their own perceived stress (*β* = −0.220, *p* < 0.01) and that parents’ perceived social support from others was negatively associated with their own perceived stress (*β* = −0.177, *p* < 0.05). Additionally, we found one partner effect where CCS perceived social support from others was negatively associated with their parents’ perceived stress (*β* = −0.164, *p* < 0.05). Once again, no significant actor or partner effect was detected from spirituality and religiosity with perceived stress for both CCS and parents.

### Moderation effects of Hispanic ethnicity

**Fig. 3:**
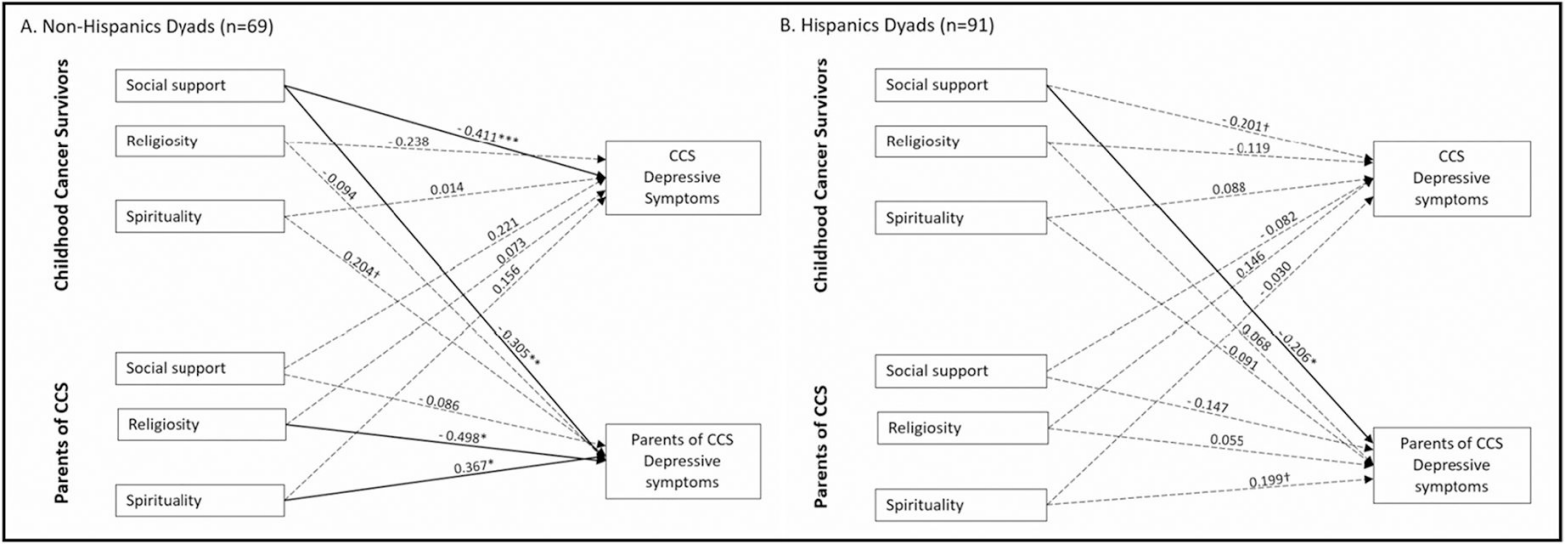
APIM model of social support, religiosity, and spirituality on CCS and parent depressive symptoms among Hispanic and non-Hispanic dyads. **a** The stratified model among Non-Hispanic Dyads (*n* = 69). **b** The stratified model among Hispanic Dyads (*n* = 91) Note. CCS: Childhood cancer survivors Figure values are standardized regression coefficients while controlling for CCS ege and treatment intensity; Solid lines represent significant estimates and dashed lines represent statistical non-significant estimates. ***P < .001. **P < .01. *P < .05. ^†^P <.10 Error covariance estimates have been omitted for ease of presentation.

**Fig. 4:**
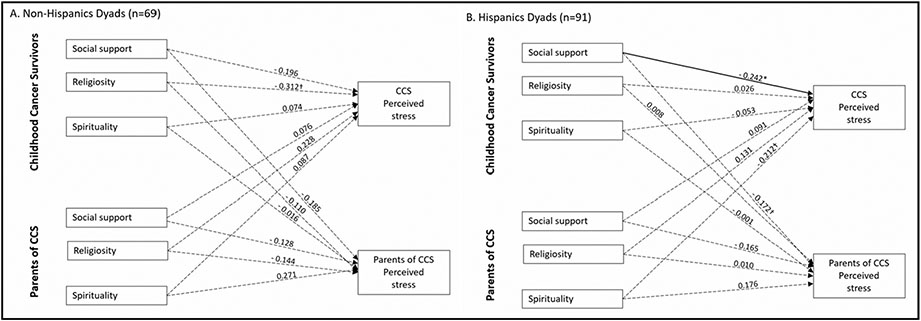
APIM model of social support, religiosity, and spirituality on CCS and parent perceived stress among Hispanic and non-Hispanic dyads. **a** The stratified model among Non-Hispanic Dyads (*n* = 69). **b** The stratified model among Hispanic Dyads (*n* = 91) Note. CCS: Childhood cancer survivors Figure values are standardized regression coefficients while controlling for CCS age and treatment intensity; Solid lines represent significant estimates and dashed lines represent statistical non-significant estimates. ***P < .001. **P < .01. *P < .05. ^†^P <.10 Error covariance estimates have been omitted for ease of presentation.

Lastly, we examined the moderating effects of parent Hispanic ethnicity on these two models (see Figs. 3 and 4). For the model related to depressive symptoms, among non-Hispanic dyads, the same actor and partner effect was found such that CCS perceived social support from others was negatively associated with their depressive symptoms (*β* = −0.411, *p* < 0.001) and their parents’ depressive symptoms (*β* = −0.305, *p* < 0.01) (see Fig. 3). Parents’ spirituality was positively associated (*β* = 0.367, *p* < 0.05) and religiosity was negatively associated (*β* = −0.498, *p* < 0.05) with their depressive symptoms. While for Hispanic dyads, we only found a significant partner effect that CCS perceived social support from others was negatively associated with their parents’ depressive symptoms (*β* = −0.206, *p* < 0.05). For the model related to perceived stress, we only found one significant actor effect finding, which was among Hispanic dyads, and CCS perceived social support from others was negatively associated with their own perceived stress (*β* = −0.242, *p* < 0.05) (see Fig. 4).

## Discussion

This study addressed a gap in the dyadic research relating to coping resources and mental health outcomes among ethnically diverse CCS and parent relationships. We found significant actor effects in CCS-parent dyads, such that social support was negatively associated with depressive symptoms and perceived stress. We also found significant partner effects that CCS social support was negatively associated with parent depressive symptoms and perceived stress. Much less is known about the dyadic relationships of Hispanic/Latino and long-term survivors, so these results provide insight into this understudied population.

Greater CCS and parent perception of social support received was associated with lower depressive symptoms and perceived stress levels, supporting our hypotheses and consistent with prior literature showing that social support is associated with parent and CCS mental health outcomes [7, 21, 22]. Higher emotional and instrumental family support is related to better psychological adjustment for CCS and parents [21]. These findings add to the existing literature by controlling for the interdependence effects of dyads; this indicates a benefit of increasing social support in CCS-parent dyad interventions.

CCS perceived social support was a significant negative predictor for parents’ depressive symptoms and perceived stress, but not vice versa. Although studies have found partner effects in the psychosocial and health outcomes of older adults cancer survivors and caregivers [41-44], our results build on past research by demonstrating that CCS perceived social support significantly affected parents’ mental health. One possible explanation for this finding is the inherent interdependent relationship between CCS and parents. Given that CCS were between the ages of 16 and 26, parents may be more susceptible to their child’s overall well-being, including that CCS perceive higher levels of social support. Furthermore, while CCS and parents experience psychological distress, parents often act as the primary supporter of CCS; with this in mind, if CCS have more social support, that might indicate less caregiver burden, ultimately explaining lower depressive symptoms and perceived stress. Moreover, there was no partner effect of parent social support on CCS mental health, which might be explained by parents’ lower levels of reported social support in comparison to CCS levels of social support. This could also indicate that there may be mediation effects of social support and psychological distress that should be explored in future research.

Contrary to our hypotheses, we did not find any actor or partner effects between religiosity (e.g., frequency of religious service attendance) or spirituality (e.g., the importance of religion or spirituality) and depressive symptoms or perceived stress. It is possible that our measures did not encompass the elements of religiosity and spirituality previously found to be associated with psychological distress. For example, Kim et al. found that the dyad’s overall level of spiritual well-being was not associated with their partners’ mental health [45]. Rather, the more the person found peace, the more likely they reported better mental health (e.g., actor effects). Similarly, another study found that higher levels of meaning/peace among metastatic lung cancer patients and their spousal caregivers were associated with their depressive symptoms and cancer distress but found no partner effects [46]. Only one study among breast cancer survivors found a partner effect in which higher levels of spirituality in spouses, which encompasses the importance of spirituality in an individual’s life and their engagement in spiritual activities, were associated with lower levels of intrusive thoughts (a subscale of emotional distress) [47]. Future studies should measure spirituality and religiosity with validated scales with multiple subscales as they may explain the lack of individual and dyadic effects. Furthermore, it is important to note that the limited literature that has explored these bidirectional relationships has been among adult spouse dyads. Therefore, the generational differences (e.g., age and different types of relationships), as well as the cultural heterogeneity of our sample, may be contributing to the null findings of these relationships.

Findings confirm the importance of cancer survivor and caregiver social support on psychological distress and suggest that there may be differences in these relationships according to Hispanic ethnicity. Specifically, Hispanic CCS perceived social support was negatively associated with their depressive symptoms. For non-Hispanic CCS, perceived social support was negatively associated with their perceived stress. Additionally, for both Hispanic and non-Hispanic CCS, perceived social support from others was associated with their parents’ depressive symptoms. These novel findings build evidence beyond individual factors, indicating dyadic coping resources such as social support are related to mental health outcomes of CCS-parent dyad post-treatment. Furthermore, these results support using the theory of dyadic illness management within our child “cancer survivor” and parent “caregiver” population, which has not been previously explored [48]. This theoretical framework suggests that dyads are an interdependent team that influence behaviors, management, and health outcomes, which ultimately supports the development of dyadic interventions that focus on psychoeducational and skill training to improve multiple aspects of quality of life [49].

### Strengths and limitations

Our study has multiple strengths. Our sample draws from a diverse region where more than half of the dyads were Hispanics. CCS included in this study had been treated for multiple cancer types representative of the most common childhood cancers in the USA. Additionally, methodological strengths include considering the interdependence nature of parent–child relationships using an APIM modeling approach, which results in more accurate findings, in contrast to other traditional regression model methods that can result in biased significance tests such as type I and II errors [39]. Lastly, CCS in this study were largely long-term survivors.

Although the current study will contribute to the growing literature on dyadic experiences, especially among the vulnerable population, this study still has limitations regarding the methodology, sampling, and measurements. The first limitation is that social support, religiosity, and spirituality measures used in this analysis were single-item, albeit each has previously been internally and externally validated. Additionally, we acknowledge that the PSS scale had poor internal consistency scores; however, our study is one of the few to look at these outcomes among dyads, and our findings demonstrate that these relationships need to be further explored. Second, we limited the covariates in our model and could not explore other clinical and demographic characteristics; as a result, there may be some residual confounding in our model results. Specifically, we recognize that time since diagnosis as a potential covariate may have played an important role in the interrelationships between coping resources and mental health but adjusting for this additional covariate made the model fit worse and did not change our primary results (meaningfully). While we carefully considered several (aforementioned) confounding factors, other potential confounding factors may need to be considered in the future research. Third, dyad members were recruited from two children’s hospitals in Los Angeles County, which means findings may not necessarily be generalizable as there is great variability among different healthcare settings. Lastly, the cross-sectional design limited analysis and findings as causality and directionality cannot be implied; future longitudinal studies examining these causal pathways may be illuminating. Additionally, since the average time since diagnosis was 7 years for CCS, future work should also explore these relationships at different phases of the cancer survivorship trajectory, including after treatment, 2 and 5 years after treatment, and longer periods of time.

In conclusion, these results suggest that we must consider interpersonal relationships and the critical cultural context that may impact these relationships within this unique and understudied population. There may be potential differences in culture, family structure, and/or class, affecting how dyads perceive/adopt coping strategies. Our findings emphasize the importance of continued psychosocial follow-up for both CCS and parents. Future research may benefit from evaluating whether interventions focusing on increasing social support post-treatment or identifying existing resources for dyads impact their mental health.

## Supplementary Material

Supplemental Material

## Data Availability

The data that support the findings of this study are available on request from the corresponding author. The data are not publicly available due to privacy or ethical restrictions.
