# Supplementary material for: Hispanic/Latinos and non-Hispanic whites’ childhood cancer survivors and parents: a dyadic analysis of coping resources and mental health
Source: J Cancer Surviv. Author manuscript; Available in PMC 2024 Aug 1. (PMC10460832; doi:10.1007/s11764-023-01339-8)
Supplement: Supplemental Material [file NIHMS1887150-supplement-Supplemental_Material.pdf]

Title: Hispanic/Latinos and non-Hispanic whites' childhood cancer survivors and parents: A dyadic analysis of coping resources and mental health

Journal: Journal of Cancer Survivorship

Authors: Carol Y. Ochoa[1], Junhan Cho[1], Kimberly A. Miller[1,2], Lourdes Baezconde-Garbanati[1], Randall Y. Chan[3], Albert J. Farias[1], Joel E. Milam[4]

Affiliations:

[1] Department of Population and Public Health Sciences, Keck School of Medicine, University of Southern California, Los Angeles, CA, USA

[2] Department of Dermatology, Keck School of Medicine, University of Southern California, Los Angeles, CA, USA

[3] Department of Pediatrics, Keck School of Medicine, University of Southern California, Los Angeles, CA, USA

[4] Department of Epidemiology and Biostatistics, Department of Medicine, University of California, Irvine, Irvine, CA, USA

Corresponding author: Carol Y. Ochoa, PhD, MPH, 2001 N. Soto Street, 3rd Floor, Room 312-30 Los Angeles, CA 90032; caroloch@usc.edu

Supplemental Figure 1. Conceptual models of dyadic influences of social support, religiosity, and spirituality on depressive symptoms.

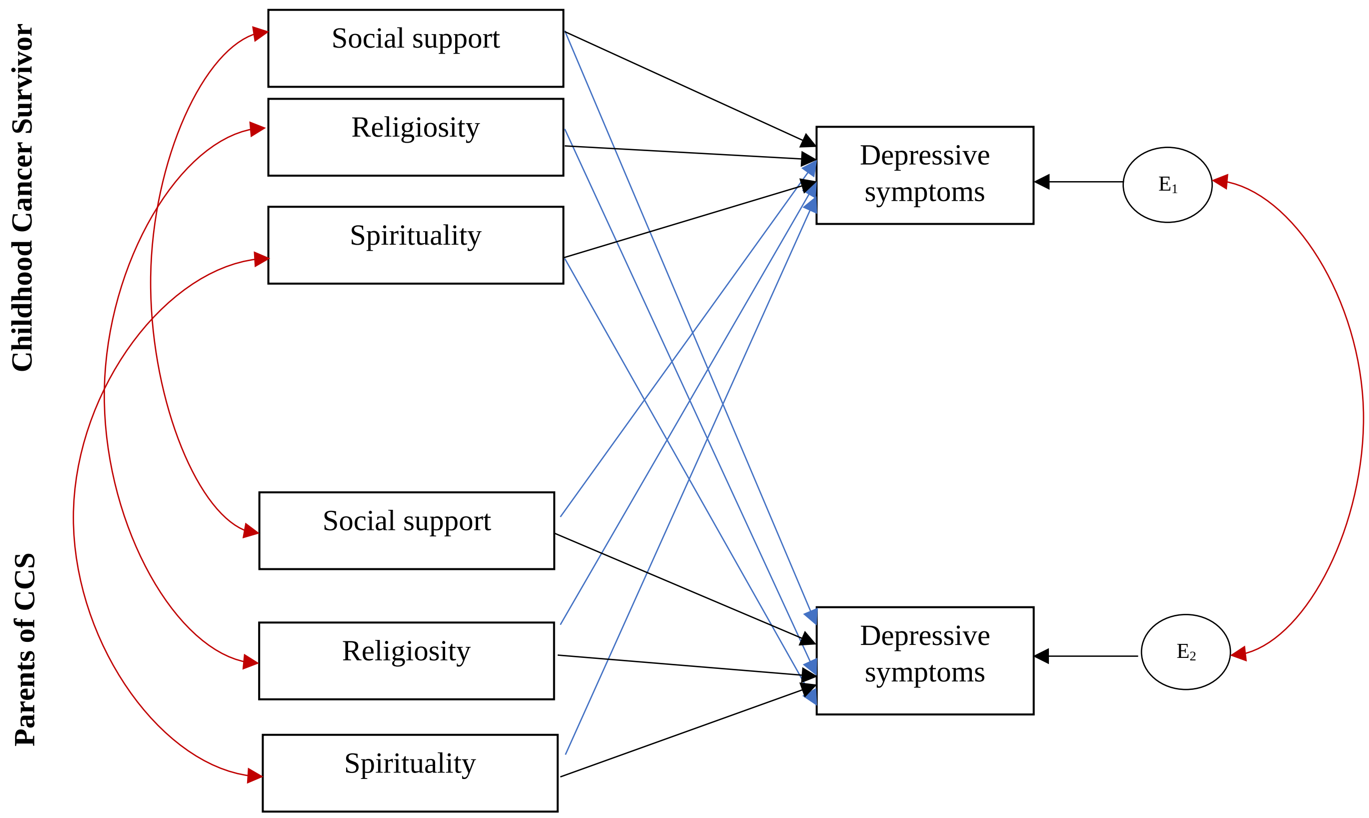

Note. Solid black lines represent actor effects, solid blue lines represent partner effects, and curved lines with a double-headed arrow represent correlated variables. For simplicity purposes, the conceptual model omitted CCS age at survey, treatment intensity, and Hispanic ethnicity as covariates for Aim 1. Additionally, the model omits illustrating the moderating effects of Hispanic ethnicity for Aim 3.

Supplemental Figure 2. Conceptual models of dyadic influences of social support, religiosity, and spirituality on perceived stress.

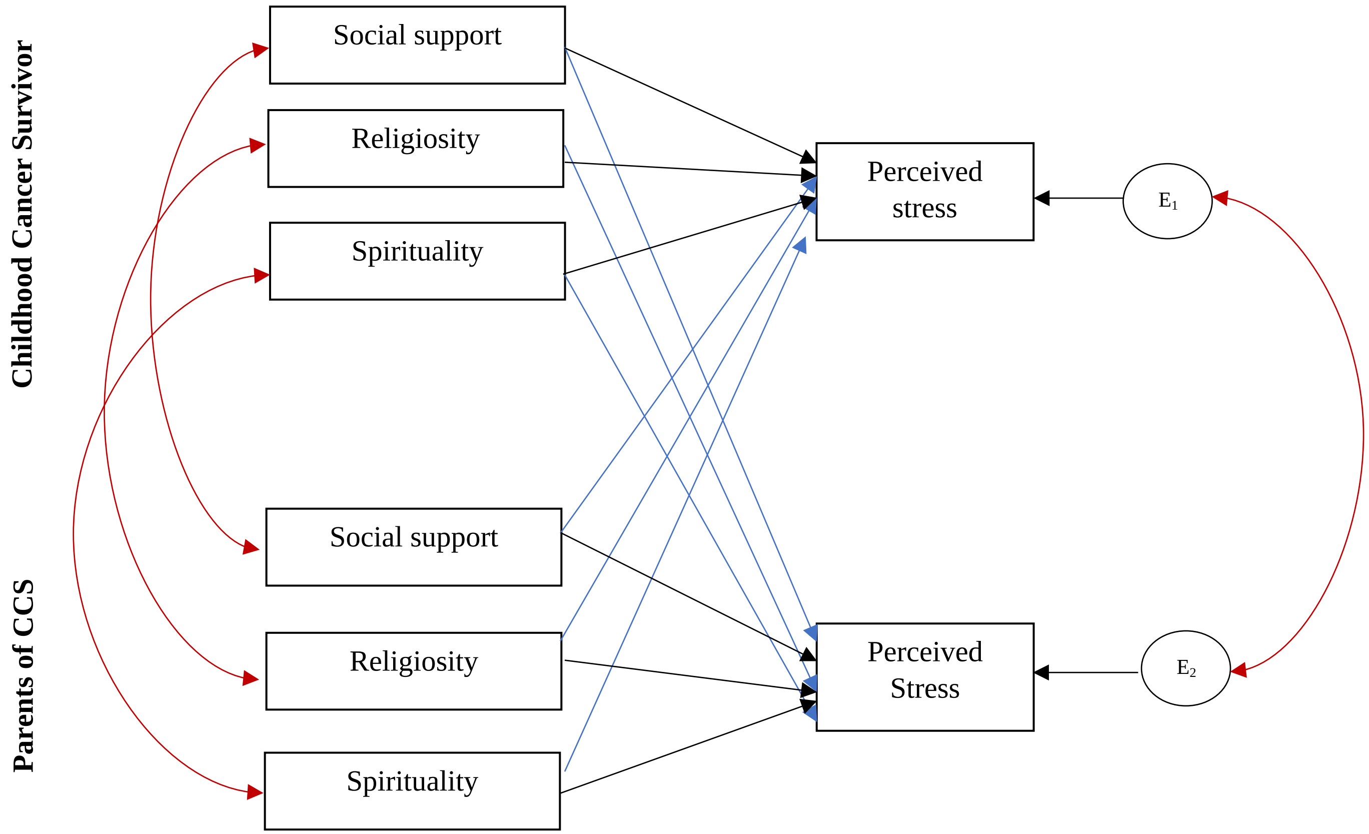

Note. Solid black lines represent actor effects, solid blue lines represent partner effects, and curved lines with a double-headed arrow represent correlated variables. For simplicity purposes, the conceptual model omitted CCS age at survey, treatment intensity, and Hispanic ethnicity as covariates for Aim 2. Additionally, the model omits illustrating the moderating effects of Hispanic ethnicity for Aim 3.

Supplemental Table 1. CCS and Parent demographic information by Hispanic ethnicity

	**Overall Dyads (n=160)**				**Hispanic Dyads (n=91)**				**Non-Hispanic Dyads (n=69)**			
**Variables**	**CCS**		**Parent**		**CCS**		**Parent**		**CCS**		**Parent**	
Age at survey (years)[a]	20.29	(2.80)	48.23	(6.15)	20.14	(2.66)	46.60	(6.00)	20.59	(3.08)	51.58	(6.57)
Gender[b]												
Male	79	49%	18	11%	46	51%	8	9%	33	48%	10	15%
Female	81	51%	141	89%	45	49%	83	91%	36	52%	58	85%
Cancer site[b]			--	--			--	--			--	--
Leukemia	48	30%			30	33%			18	26%		
Brain	27	17%			12	13%			15	22%		
Bone	10	6%			7	8%			3	4%		
Lymphoma	31	19%			17	19%			14	20%		
Other	44	28%			25	27%			19	28%		
Time since diagnosis (years)[a]	7.41	(2.13)	--	--	7.33	(2.19)	--	--	7.57	(2.03)	--	--
Treatment intensity[b]			--	--			--	--			--	--
1	16	10%			8	9%			8	12%		
2	52	33%			32	35%			20	29%		
3	76	48%			40	44%			36	53%		
4	15	9%			11	12%			4	6%		
Educational Level[b]												
<12 years	61	38%	55	35%	37	41%	51	57%	24	35%	4	6%
High school graduate/GED	29	18%	21	13%	22	24%	15	17%	7	10%	6	9%
>12 years	69	43%	82	52%	31	34%	24	27%	38	55%	58	68%
Socioeconomic status[b]	--	--			--	--			--	--		
Low			75	47%			67	74%			8	12%
Middle/High			85	53%			24	26%			61	88%

Note: [a] Mean and standard deviation; [b] number and percentage.

Supplemental Table 2. Mean, standard deviation, and correlations of parent and childhood cancer survivors study variables and covariates for overall sample

	1	2	3	4	5	6	7	8	9	10	11	12	13
Mean	1.61	2.49	1.31	15.63	5.67	1.24	1.87	1.53	14.35	5.29	2.57	20.34	0.56
SD	0.62	1.38	0.71	12.71	3.54	0.73	1.42	0.5	11.65	3.16	0.80	2.85	0.50
Paired t-test[a]	<0.001	<0.001	0.0006	0.3812	0.3089	--	--	--	--	--	--	--	--
1. Parent spirituality	1.00												
2. Parent religiosity	0.60***	1.00											
3. Parent social support	-0.16*	0.03	1.00										
4. Parent depressive symptoms	0.19*	0.08	-0.33***	1.00									
5. Parent perceived stress	0.18*	0.07	-0.27***	0.78***	1.00								
6. CCS spirituality	0.30***	0.28***	-0.09	0.13†	0.05	1.00							
7. CCS religiosity	0.26***	0.56***	0.12	-0.01	-0.04	0.56***	1.00						
8. CCS social support	-0.04	-0.03	0.31***	-0.30***	-0.22**	-0.15†	0.02	1.00					
9. CCS depressive symptoms	0.11	0.10	-0.09	0.33***	0.27***	0.07	-0.03	-0.29***	1.00				
10. CCS perceived stress	0.05	0.08	-0.02	0.15†	0.15†	0.03	-0.03	-0.22***	0.73***	1.00			
11. CCS treatment Intensity	0.21**	-0.03	0.04	0.01	-0.03	0.06	0.002	0.001	0.07	0.06	1.00		
12. CCS age	-0.04	-0.10	0.06	-0.04	-0.08	-0.02	-0.10	-0.05	0.03	0.02	-0.09	1.00	
13. Hispanic ethnicity	0.25**	0.27***	-0.19*	0.23**	0.09	0.17*	0.11	-0.14†	0.10	0.10	0.04	-0.08	1.00

Abbreviations. CCS: Childhood cancer survivors; SD: standard deviation

Notes: Depressive symptom values are self-reported depressive symptom total scores on the Center for Epidemiological Studies Depression Scale; and Perceived stress values are self-reported total scores on Perceived Stress Scale for parents and childhood cancer survivors, respectively.

*** p<0.001; ** p<0.01; * p<0.05; † p<0.10

[a] Paired sample t-test between the same independent and dependent variables of parent and CCS (e.g., parent depressive symptoms and CCS depressive symptoms)

Supplemental Table 3. Distribution of predictors and outcomes by Hispanic ethnicity

	**Hispanic Dyads (n=91)[a]**		**Non-Hispanic Dyads[a] (n=69)**	
	CCS	Parent	CCS	Parent
Predictors				
Social support	1.45 (0.58)†	1.19 (0.71)*	1.62 (0.60)†	1.46 (0.70)*
Religiosity	2.01 (1.49)	2.79 (1.30)*	1.69 (1.30)	2.03 (1.38)*
Spirituality	1.35 (0.72)*	1.74 (0.49)*	1.10 (0.73)*	1.43 (0.72)*
Outcomes				
Depressive symptoms	15.35 (11.97)	18.20 (13.00)*	13.06 (11.18)	12.39 (11.64)*
Perceived stress	5.57 (2.91)	5.95 (3.53)	4.93 (3.45)	5.32 (3.56)

[a] Comparing CCS predictors or outcomes by Hispanic ethnicity and parent predictors or outcomes by Hispanic ethnicity.
*p<0.05; †p<0.10
